# Supplementary material for: Effect of 1,2,4,5-Benzenetetracarboxylic Acid on Unsaturated Poly(butylene adipate-co-butylene itaconate) Copolyesters: Synthesis, Non-Isothermal Crystallization Kinetics, Thermal and Mechanical Properties
Source: Polymers (Basel). 2020 May 19;12(5):1160. doi: 10.3390/polym12051160 (PMC7285232; doi:10.3390/polym12051160)
Supplement: Supplementary file 1 [file polymers-12-01160-s001.docx]

**Effect of 1,2,4,5-benzenetetracarboxylic acid on unsaturated poly(butylene adipate-co-butylene itaconate) copolyesters: Synthesis, non-isothermal crystallization kinetics, thermal and mechanical properties**

Chin-Wen Chen, Te-Sheng Hsu, Kuan-Wei Huang, and Syang-Peng Rwei*

Institute of Organic and Polymeric Materials, Research and Development Center of Smart Textile Technology, National Taipei University of Technology,

No. 1, Sec. 3, Chung-Hsiao East Road., Taipei, 10608, Taiwan (R.O.C.)

******************************************************************************

**Supporting Information**

**Table S1** The composition of the calculated C=C bond of itaconic acid, chemical shifts (in ppm) and the integral ratio (the value in brackets) of ^1^H NMR spectra for PBABI copolyesters……………………………..2

**Figure S1.** ^1^H NMR spectra of PBABI copolyesters at (a) BA/BI = 100/0, (b) BA/BI = 95/5, (c) BA/BI = 85/15, and (d) BA/BI = 80/20…..……………………………………………………………………………...3

**Figure S2.** Relative crystallinity (X(t)) as a function of time for PBABI copolyesters with a BA/BI content in (a) BA/BI = 100/0, (b) BA/BI = 95/5, (c) BA/BI = 90/10, (d) BA/BI = 85/15, and (e) BA/BI = 80/20 at different cooling rates……………………………………………………………………………………………………5

**Figure S3.** Avrami polt of log{-ln[1-X(t)]} versus log(t) for PBABI copolyesters with a BA/BI content in (a) BA/BI = 100/0, (b) BA/BI = 95/5, (c) BA/BI = 90/10, (d) BA/BI = 85/15, and (e) BA/BI = 80/20 at different cooling rates……………………………………………………………………………………………………6

**Figure S4.** Plot of log($\emptyset$) versus log(t) for PBABI copolyesters with a BA/BI content in (a) BA/BI = 100/0, (b) BA/BI = 95/5, (c) BA/BI = 90/10, (d) BA/BI = 85/15, and (e) BA/BI = 80/20 at a range of crystallinity in 0.2–0.8 during non-isothermal crystallization based on Mo's equation. ………………………………………7

**Figure S5.** Plot of ln($\emptyset$/T_p_^2^) vs. 1/T_p_ for PBABI copolyesters at different BA/BI contents. …………….……….8

**Figure S6.** Plot of ln($\emptyset$) vs. 1/(2.3T_p_^2^) for PBABI copolyesters. ……………….………………………….….8

**Figure S7.** ^1^H NMR spectra of PBABI copolyesters at (a) BA/BI = 90/10 – 0.05, (b) BA/BI = 90/10 – 0.1, and (c) BA/BI = 90/10 – 0.2. ……………………………………………………….………………………..10

**Figure S8.** FT-IR spectra of PBABI copolyesters with different concentrations of BTCA. ………….……..10

**Figure S9.** XRD patterns of BA/BI = 90/10 of PBABI copolyesters with different concentrations of BTCA..11

**Table S1.** The composition of the calculated C=C bond of itaconic acid, chemical shifts (in ppm) and the integral ratio (the value in brackets) of ^1^H NMR spectra for PBABI copolyesters.

| **Sample** | **Feed Ratio** | **Calculated Ratio** | **H_1_** | **H_2_** | **H_3_** | **H_4_** | **H_5_** | **H_6_** | **H_7_** | **H_8_** |
| --- | --- | --- | --- | --- | --- | --- | --- | --- | --- | --- |
| **BA/BI = 100/0** | 0 | 0 | 1.877  (0.993) | 1.960  (0.964) | 2.643  (1.000) |  | 4.404  (0.986) |  |  | 8.320  (0.007) |
| **BA/BI = 95/5** | 5 | 4.07 | 2.124  (0.999) | 2.206  (0.996) | 2.889  (1.000) | 3.912  (0.021) | 4.648  (0.984) | 6.054  (0.030) | 6.319  (0.012) | 6.913  (0.012) |
| **BA/BI = 90/10** | 10 | 4.21 | 1.947  (1.002) | 2.031  (0.961) | 2.712  (1.000) | 3.730  (0.033) | 4.466  (0.952) | 6.142  (0.016) | 6.737  (0.016) | 7.131  (0.009) |
| **BA/BI = 85/15** | 15 | 8.48 | 1.894  (1.004) | 1.983  (1.080) | 2.663  (1.000) | 3.689  (0.067) | 4.430  (1.046) | 6.095  (0.038) | 6.689  (0.037) | 7.084  (0.019) |
| **BA/BI = 80/20** | 20 | 9.53 | 2.106  (1.008) | 2.195  (1.040) | 2.865  (1.000) | 3.889  (0.058) | 4.630  (1.021) | 6.031  (0.062) | 6.295  (0.040) | 6.895  (0.037) |


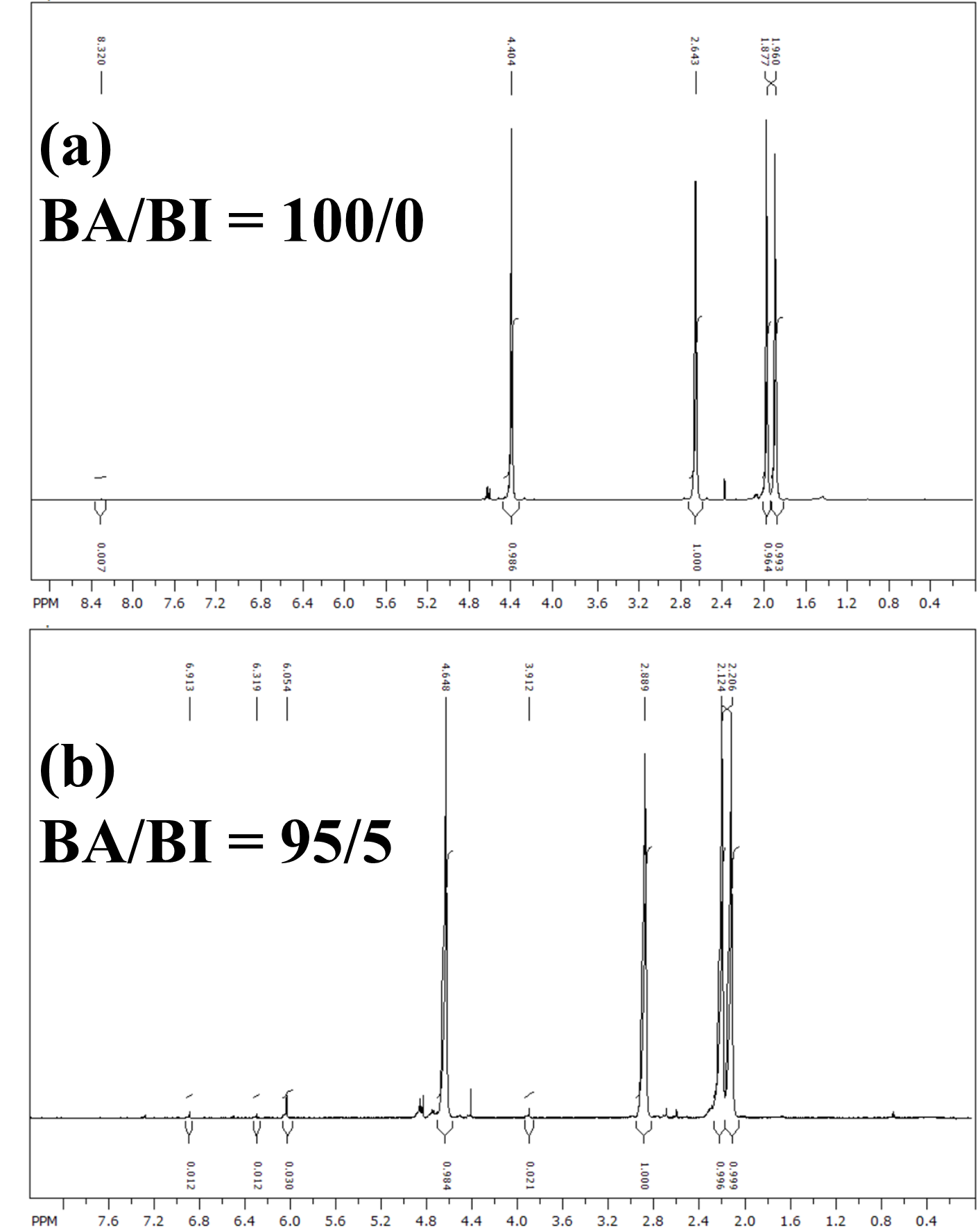


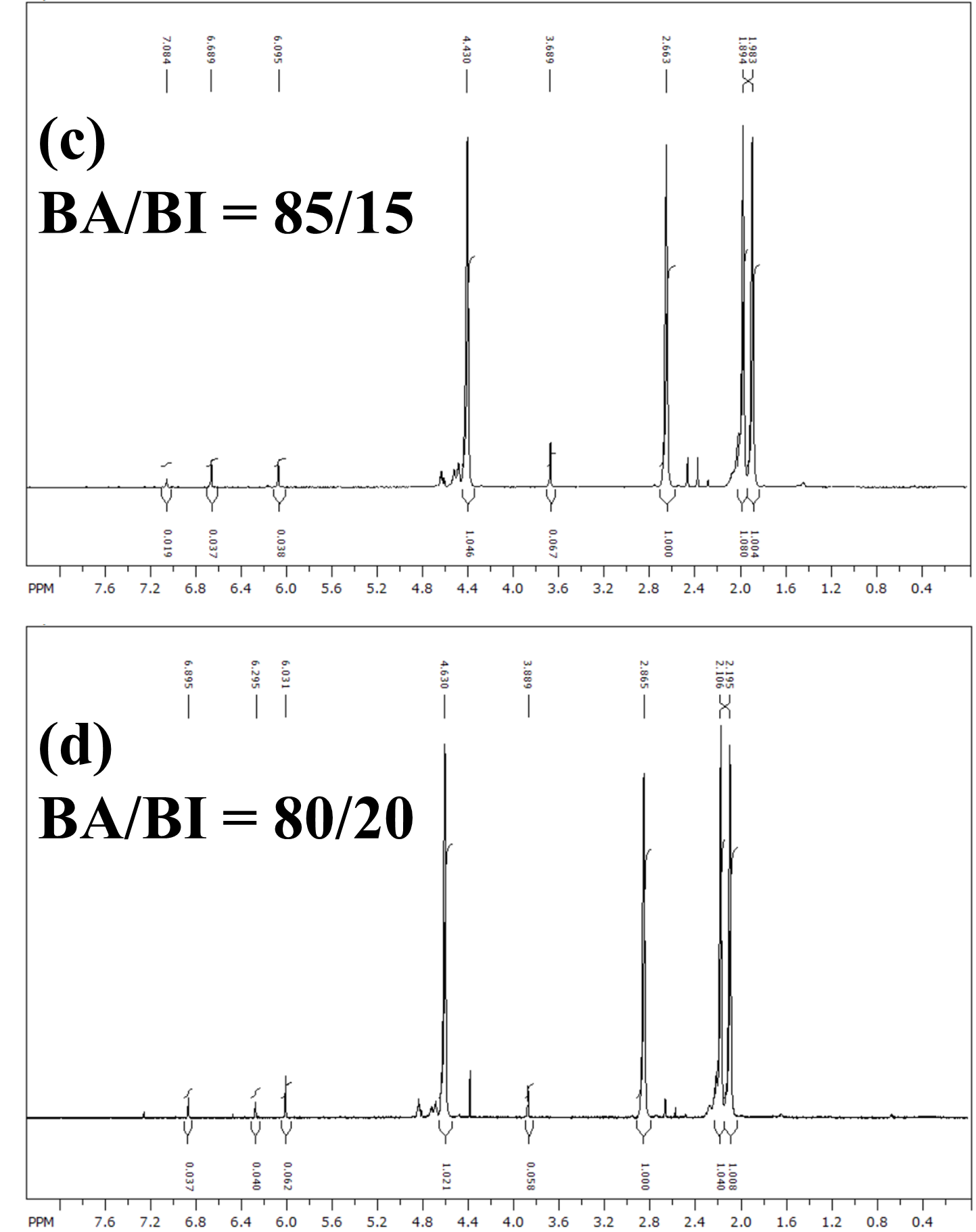


**Figure S1.** ^1^H NMR spectra of PBABI copolyesters at (a) BA/BI = 100/0, (b) BA/BI = 95/5, (c) BA/BI = 85/15, and (d) BA/BI = 80/20.

**
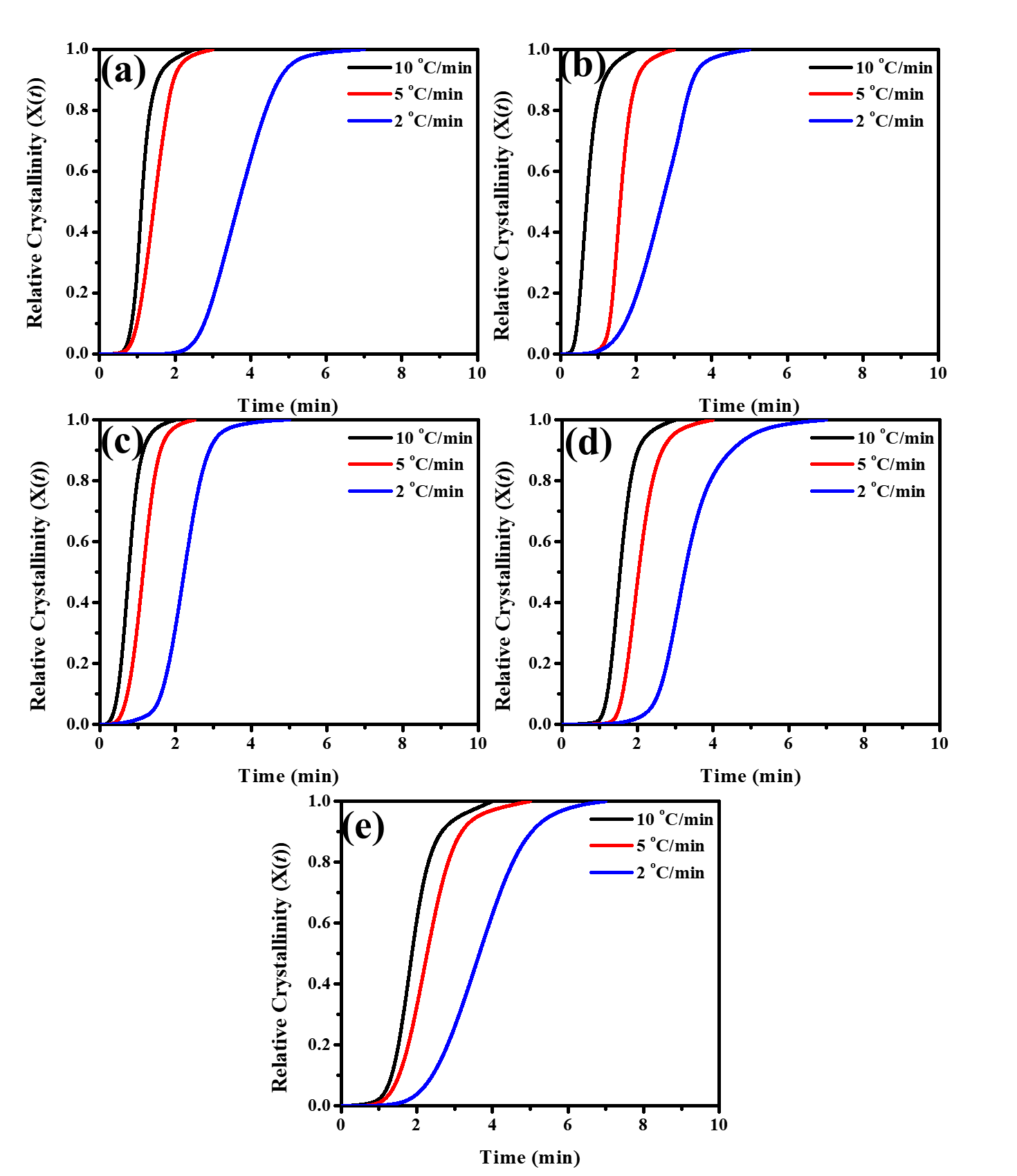
**

**Figure S2.** Relative crystallinity (X(*t*)) as a function of time for PBABI copolyesters with a BA/BI content in (a) BA/BI = 100/0, (b) BA/BI = 95/5, (c) BA/BI = 90/10, (d) BA/BI = 85/15, and (e) BA/BI = 80/20 at different cooling rates.


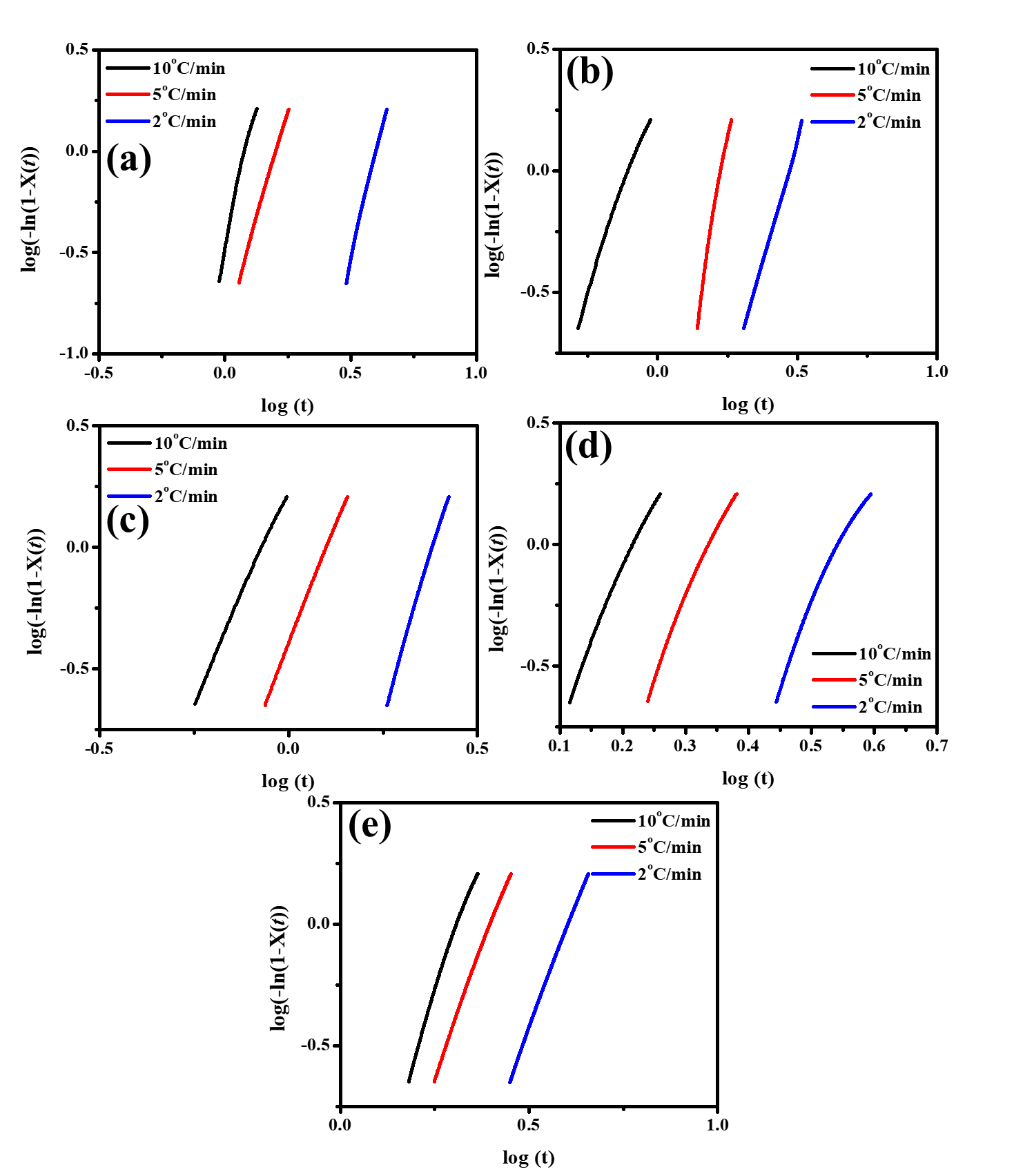


**Figure S3.** Avrami polt of log{-ln[1-X(t)]} versus log(t) for PBABI copolyesters with a BA/BI content in (a) BA/BI = 100/0, (b) BA/BI = 95/5, (c) BA/BI = 90/10, (d) BA/BI = 85/15, and (e) BA/BI = 80/20 at different cooling rates.

*
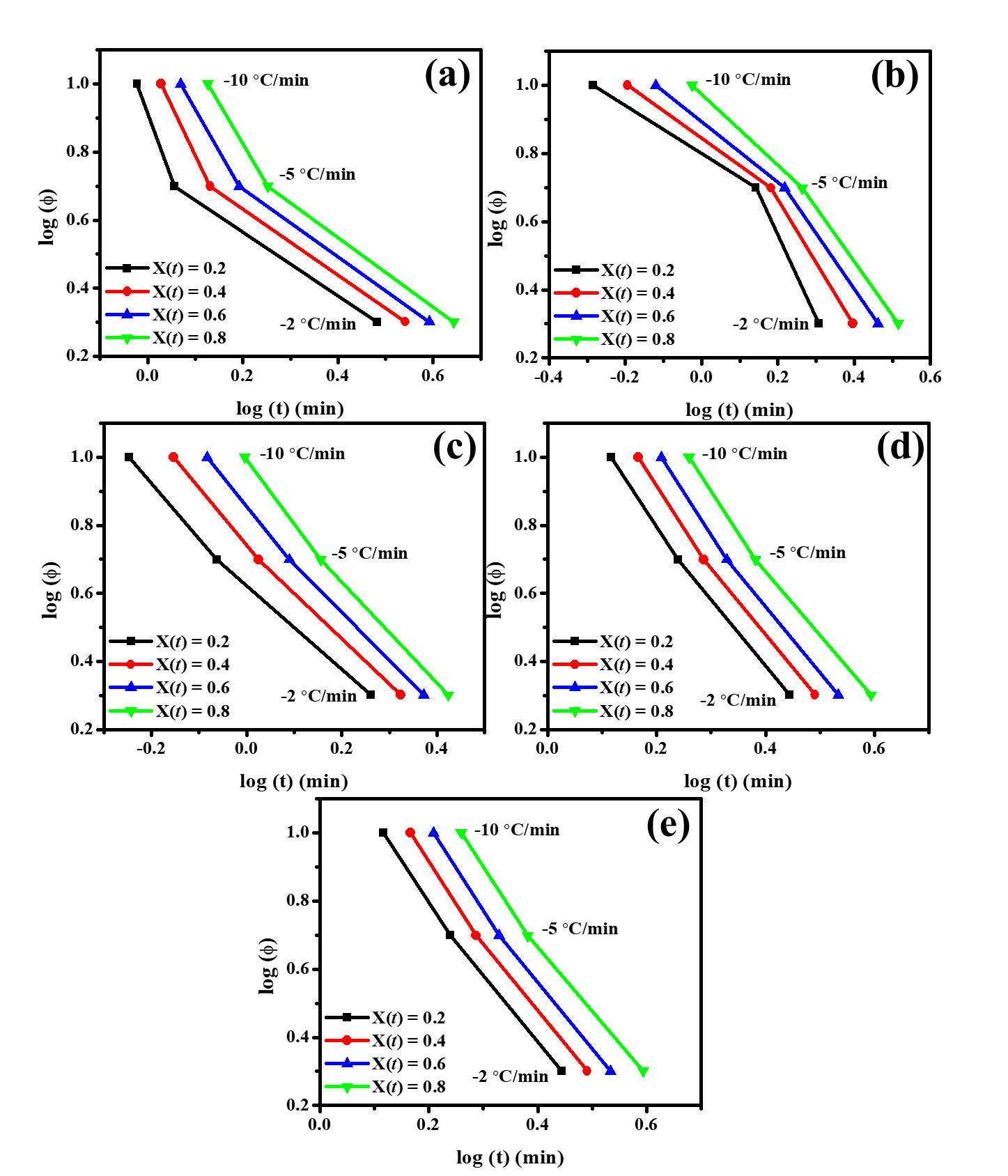
*

**Figure S4.** Plot of log($\emptyset$) versus log(t) for PBABI copolyesters with a BA/BI content in (a) BA/BI = 100/0, (b) BA/BI = 95/5, (c) BA/BI = 90/10, (d) BA/BI = 85/15, and (e) BA/BI = 80/20 at a range of crystallinity in 0.2–0.8 during non-isothermal crystallization based on Mo's equation.

**Figure S5.** Plot of ln($\emptyset$/T_p_^2^) vs. 1/T_p_ for PBABI copolyesters at different BA/BI contents.

**Figure S6.** Plot of ln($\emptyset$) vs. 1/(2.3T_p_^2^) for PBABI copolyesters.


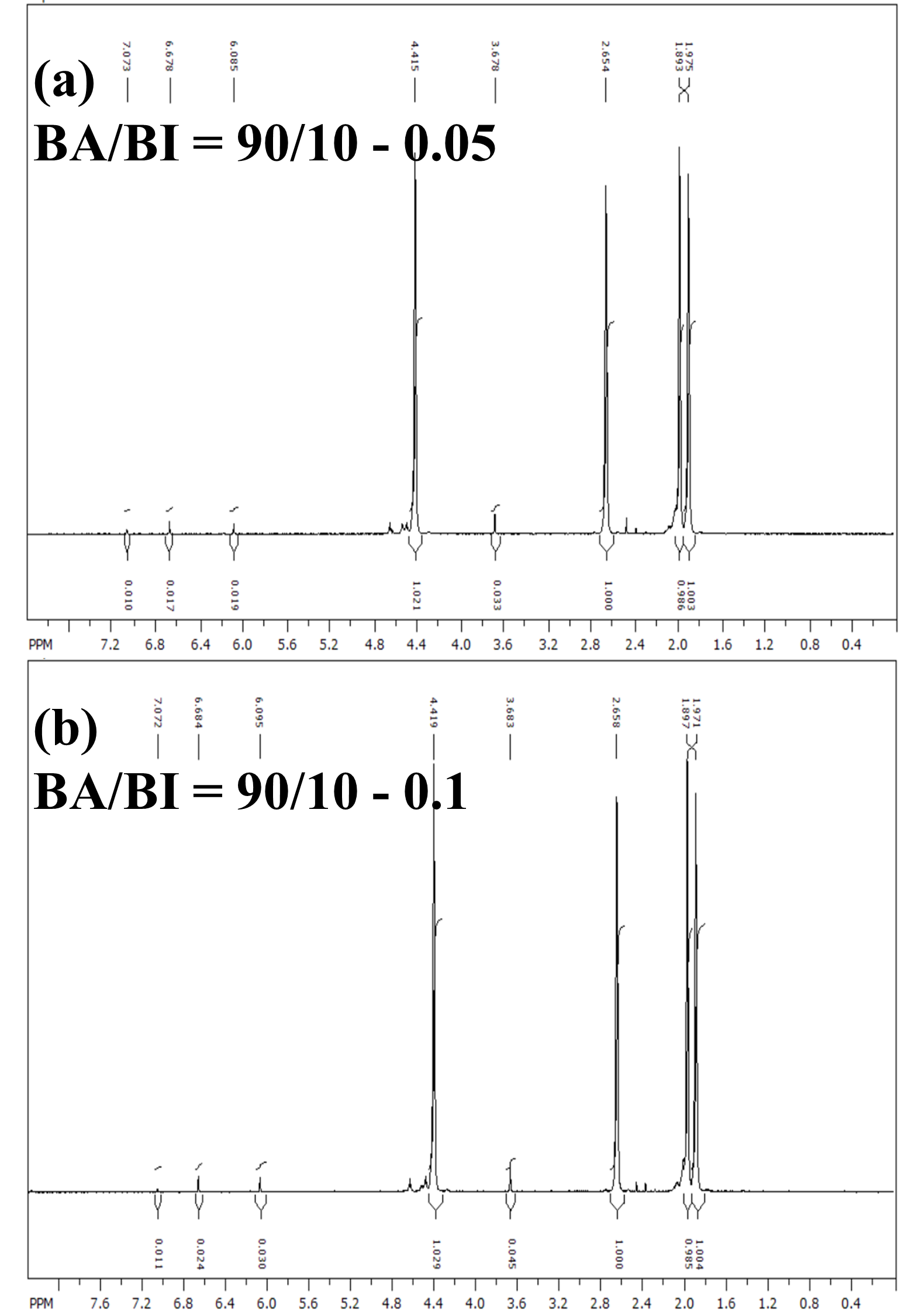


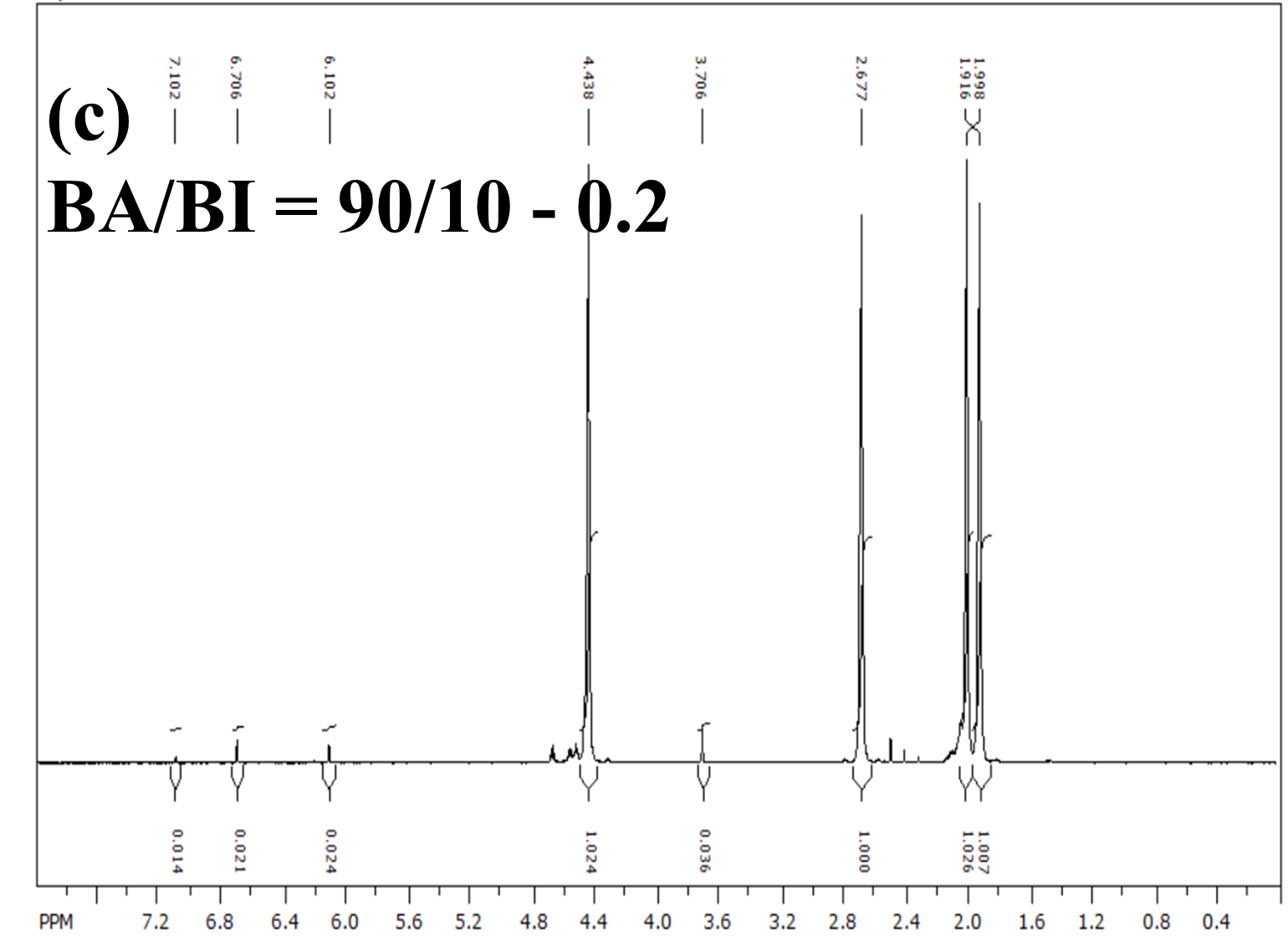


**Figure S7.** ^1^H NMR spectra of PBABI copolyesters at (a) BA/BI = 90/10 – 0.05, (b) BA/BI = 90/10 – 0.1, and (c) BA/BI = 90/10 – 0.2.

**Figure S8.** FT-IR spectra of PBABI copolyesters with different concentrations of BTCA.

**
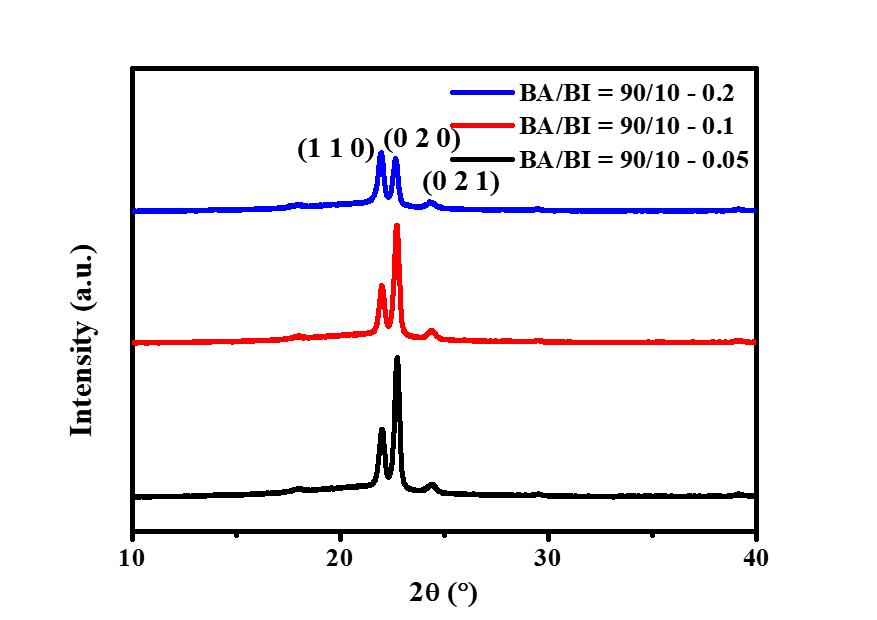
**

**Figure S9.** XRD patterns of BA/BI = 90/10 of PBABI copolyesters with different concentrations of BTCA.
